# Surgical outcomes of unilateral lateral rectus recession versus recess-resect in children with convergence insufficiency type intermittent exotropia

**DOI:** 10.1038/s41598-022-12664-w

**Published:** 2022-05-21

**Authors:** Yoon Kyung Jang, Seok Hyun Bae, Dong Gyu Choi

**Affiliations:** Department of Ophthalmology, Kangnam Sacred Heart Hospital, Hallym University College of Medicine, 665 Shiheongdae-ro, Seoul, 07442 South Korea

**Keywords:** Diseases, Medical research

## Abstract

To determine the efficacy of unilateral lateral rectus recession (ULR) for convergence insufficiency-type intermittent exotropia (CI-type IXT), we compared surgical outcomes following ULR and recess‒resect (RR) procedures for CI-type IXT. In this retrospective study, medical records of 57 children who underwent ULR (n = 30) or RR (n = 27) for CI-type IXT of less than 25 PD at distance with a postoperative follow-up of 6 months or more were reviewed. Surgical success was defined as an alignment between 10 PD exodeviation and 5 PD esodeviation at distance and near fixation. The postoperative exodeviation showed no significant difference between the two groups at the last follow-up. A significant reduction in the mean near-distance difference was achieved postoperatively in both groups: from 5.4 PD preoperatively to 2.5 at last follow-up after ULR, and from 8.2 to 2.4 after RR (both *p* = 0.001). However, this difference between ULR and RR was not statistically significant (*p* > 0.05). The success rate at the last follow-up was 63.3% for ULR and 70.4% for RR (*p* = 0.574). ULR was found to be an effective treatment for CI-type IXT, with similar surgical outcomes to RR.

## Introduction

Convergence insufficiency-type intermittent exotropia (CI-type IXT) is characterized by a greater exodeviation at near than at distance fixation, with a difference of 5‒15 prism dioptres (PD) or more^1–3^. CI-type IXT is thought to be associated with insufficient fusional convergence, a low accommodative convergence to accommodation ratio, and low accommodative amplitudes^4,5^.

Various surgeries are recommended for CI-type exotropia, including bilateral medial rectus muscle (MR) resections with or without the slanting procedure, bilateral lateral rectus muscle (LR) recession with or without the slanting procedure, LR recession with MR resection (RR), unilateral medial rectus resection with or without the slanting procedure, and MR resection with an adjustable suture^4,6–11^. The success rates of surgical procedures for CI-type exotropia have been reported to range from 18 to 92%^1,6,8–11^.

Unilateral LR recession (ULR) for IXT of the basic or divergence excess type has been reported in many studies. It was found to be a safe and effective treatment for small to moderate angle exotropia^12–16^. On the other hand, to the best of our knowledge, no study has investigated the surgical outcomes of ULR in CI-type exotropia because most surgeons are concerned about the unproven hypothesis that LR recession(s) would affect distance deviation more than near deviation^11,17^. Archer^18^ investigated how the choice between medial and lateral rectus muscle surgery affects distance-near incomitance, and concluded that the apparent greater effect of MR surgery on near deviation and LR on distance was probably an artifact. Therefore, the purpose of this study was to compare the outcomes of ULR and RR to evaluate the efficacy of ULR for CI-type IXT measuring less than 25 PD.

## Methods

### Subject recruitment

We retrospectively analyzed the medical records of 57 patients who underwent ULR (ULR group, n = 30) or RR (RR group, n = 27) for CI-type IXT measuring less than 25 PD at distance, with poor control between 2010 to 2017, with a postoperative follow-up period of 6 months or more. The control of exodeviation was scaled as good, fair or poor.: Good control was defined as “fusion breaks only after cover testing at distance fixation and resumes rapidly without need for blinking or refixation”, fair control as “subject blinks or refixates to control deviation after disruption with cover testing at distance fixation”, and poor control as “subject breaks spontaneously without any form of fusion disruption or does not spontaneously regain alignment despite blinking or refixation”^19^. All patients fulfilling the inclusion criteria were enrolled in this study. The demographic data of the patients are shown in Table 1. Our study was performed according to the tenets of the Declaration of Helsinki and was approved by the Institutional Review Board of Hallym University Medical Center (IRB No. 2020-02-012), with exemption from the need to obtain informed consent because of the retrospective study design and use of anonymized clinical data.

**Table 1:** Surgical dosage based on near exodeviation angles.

PD	Unilateral LR recession (mm)	LR recess (mm)/MR resect (mm)
20	9.0	5.0/4.0
25	10.0	6.0/5.0
30	11.0	7.0/5.5

*PD* prism diopters, *LR* lateral rectus, *MR* medial rectus.

CI-type IXT was classified based on the near-distance (N-D) exodeviation difference, which was defined as exodeviation greater by 5 PD or more at near than at distance fixation. Patients with paralytic or restrictive strabismus, previous ocular or strabismus surgery, chromosomal anomalies, or systemic disorders, such as congenital anomalies or neurologic disorder, were excluded from the study.

### Preoperative evaluation

We reviewed the following preoperative characteristics: age at onset and at surgery, sex, best-corrected visual acuity, follow-up duration, pre- and postoperative deviations at distance and near fixation, presence of lateral gaze incomitance, associated strabismus, such as dissociated vertical deviation, vertical deviation, and oblique muscle dysfunction; sensory status using the Titmus stereotest (Stereo Optical Co., Inc., Chicago, IL, USA); and Worth-4-Dot test (Richmond Products, Albuquerque, NM, USA).

All patients underwent complete ophthalmological and orthoptic evaluations prior to surgery. Cycloplegic refraction examinations were performed with 1% cyclopentolate hydrochloride (Cyclogyl, Alcon Lab. Inc., Fort Worth, TX, USA) and 1% tropicamide (Mydriacyl, Alcon Lab. Inc.). The angles of deviation were measured by the prism and alternate cover test at distance (6 m) and near (33 cm), using accommodative targets with their best optical correction. Vertical deviation was defined as hypertropia/hypotropia more than 5 PD in the primary position. Lateral gaze incomitance was defined as a decrease in the exo-angle of 20% or more in the right or left gaze, as compared with that in the primary position. Sensory status was evaluated in all patients, if possible: when a patient saw four dots in the Worth four-dot test at distance, the result was recorded as “fusion,” and stereoacuity of 100 s of arc or better using the Titmus stereotest at near was defined as “good stereopsis.”

### Surgery

All surgeries were performed under general anesthesia, using the standard limbal incision without any hangback or adjustable sutures, by a single surgeon (DGC) who had no preference for either the ULR or RR procedure. The operation was performed on the non-dominant (non-fixating) eye if the patient had a dominant eye, and on either eye if they had an alternate fixation. The surgical dosages were based on the angles of near exodeviation shown in Table 1.

### Postoperative management

Alignment at distance and near fixation in the primary position was measured in all patients at 1, 3, 6, 12, and 24 months postoperatively and at their last follow-up visit. Subjective diplopia and postoperative lateral gaze incomitance (defined as esodeviation greater by 5 PD or more in the lateral gaze of the operated eye than in the primary position) were checked on every visit. If the patients had diplopia or esodeviation postoperatively, alternate full-time patching was prescribed and was continued until the diplopia or esodeviation disappeared.

### Main outcome measures

The main outcomes were postoperative alignment at distance and near fixation, the N-D difference, and surgical success rates, which were compared between the ULR and RR groups at each visit. Surgical success was considered as alignment between exodeviation of < 10PD and esodeviation of < 5PD at distance and near fixation. We analyzed the reduction in the N-D difference at the last visit after the ULR and RR procedure, respectively, which was also compared between the two groups. Additionally, the duration of diplopia due to esodeviation (overcorrection) in the early postoperative period, particularly at distance fixation, was assessed.

### Statistical analysis

Statistical analyses were performed using SPSS software, version 24 (SPSS Inc, Chicago, IL, USA). Mann–Whitney U test and Fisher’s exact test were used to compare the preoperative characteristics between the groups. Fisher’s exact test was used to compare the success rates between two groups. The Mann‒Whitney U test was used to compare the preoperative and postoperative angles of deviation at distance and near, and the differences and N-D difference between them. A statistically significant difference was considered if the *P* value was less than 0.05. The plus sign in the deviating angle indicates exodeviation and the minus sign indicates esodeviation.

## Results

The mean follow-up period after surgery was 30.2 ± 37.0 months (range 6–75 months) in ULR, 30.0 ± 35.7 months (range 6–115 months) in RR (*p* = 0.626). The preoperative mean exodeviation was not significantly different between ULR (18.0 ± 2.5 PD) and RR (18.9 ± 2.4) on distant fixation (*p* = 0.106), however, there were significantly larger angles in RR (27.1 ± 2.5) than in ULR (23.4 ± 2.8) on near fixation (*p* < 0.05). There was a statistically significant difference in preoperative mean N-D difference between the two groups (*p* = 0.001), where ULR showed lower results (5.4 ± 1.2PD) compared to RR (8.2 ± 4.0) (Table 2).

**Table 2:** Demographic data of subjects.

	ULR group (n = 30)	RR group (n = 27)	*p* value
Age at surgery (years)	9.3 ± 3.0	9.0 ± 3.4	0.614*
Sex (male/female)	13/17	13/14	0.716†
**Exodeviation (PD)**			
At distance	18.0 ± 2.5	18.9 ± 2.4	0.106*
At near	23.4 ± 2.8	27.1 ± 2.5	0.000*
Near-distance difference (PD)	5.4 ± 1.2	8.2 ± 4.0	0.001*
Lateral incomitance [n (%)]	0 (0.0)	1 (3.7)	0.474†
**Associated strabismus**			
DVD [n (%)]	2 (6.7)	1 (3.7)	1.000†
Vertical deviation [n (%)]	2 (6.7)	8 (29.6)	0.035†
Oblique muscle dysfunction [n (%)]	7 (23.3)	8 (29.6)	0.590†
**Preoperative sensory status**			
Good stereopsis [n (%)] (100 arcsec or better in Titmus test)	26/28 (92.9%)	22/25 (88.0%)	0.658†
Fusion on Worth-4-dot test [n (%)]	17/28 (60.7%)	15/25 (60.0%)	0.958†
Postoperative follow-up duration (months)	30.2 ± 37.0	30.0 ± 35.7	0.626*

*PD* prism dioptres, *DVD* dissociated vertical deviation, *ULR group* patients who underwent unilateral lateral rectus recession, *RR group* patients who underwent unilateral lateral rectus recession-medical rectus resection.

*Mann–Whitney U test; †Fisher’s exact test.

**Figure 1:**
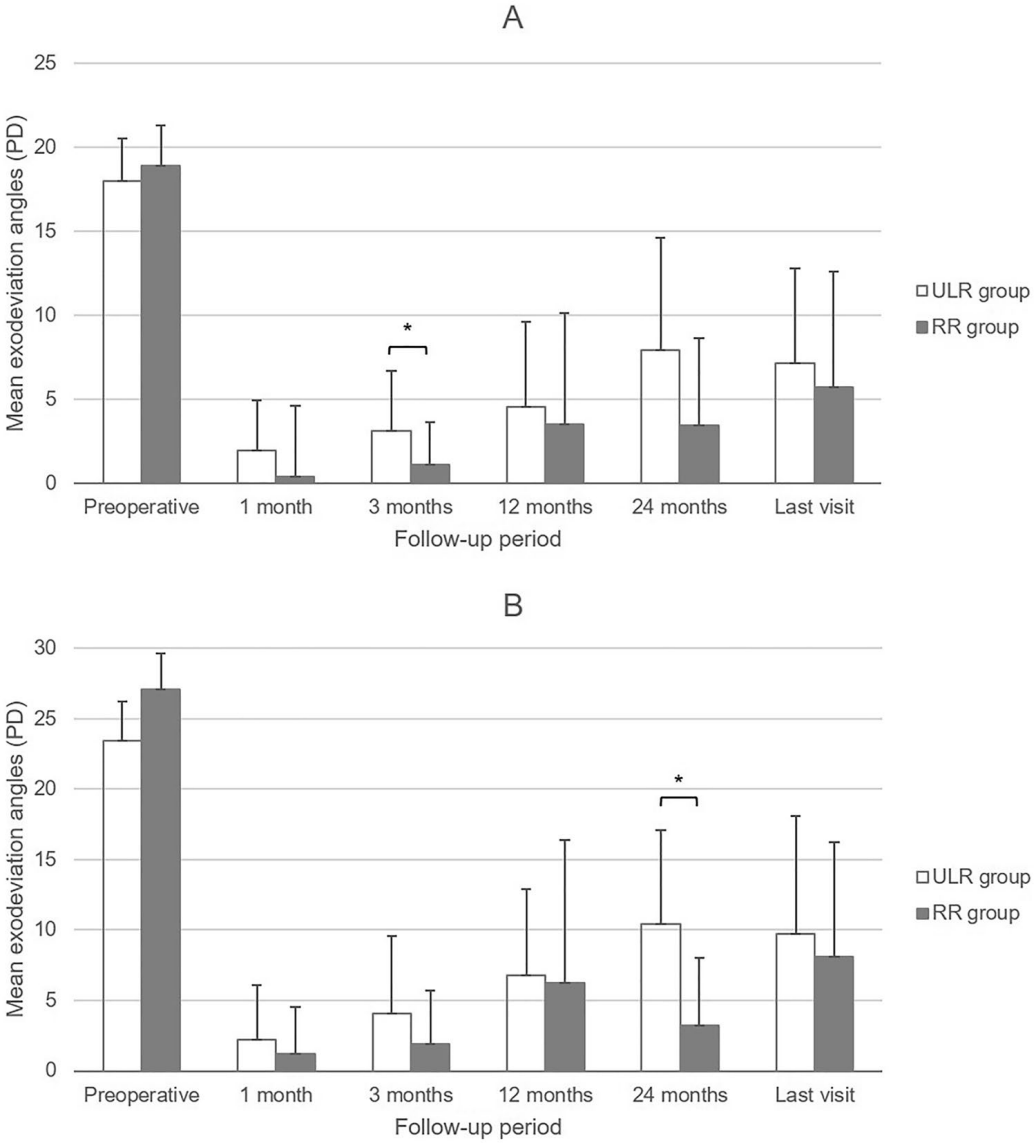
The mean pre- and postoperative exodeviation angles (PD) (**A**) at distance, and (**B**) at near in ULR group (n = 30) and RR group (n = 27). ULR group = patients who underwent unilateral lateral rectus recession. RR group = patients who underwent unilateral lateral rectus recession-medical rectus resection. PD = prism diopters. *Statistically significant between the 2 groups in Mann–Whitney U test (*P* < 0.05).

The postoperative exodeviation showed no significant difference between the two groups from 1 month postoperatively to the last follow-up (*p* > 0.05), except at 3 months at distance and 24 months at near. Mean distance exodeviation at 3 months (3.1 ± 3.6 PD in ULR and 1.1 ± 2.5 in RR, *p* = 0.047) and near exodeviation at 24 months (10.4 ± 6.7 and 3.2 ± 4.8, *p* = 0.015) were significantly larger in ULR than in RR. The mean exodeviation at the last visit was 7.1 ± 5.7 PD and 5.7 ± 6.9 at distance (*p* = 0.265) and 9.7 ± 8.4 and 8.1 ± 8.1 at near (p = 0.435) in the ULR and RR groups, respectively; there were no significant differences between the two groups (Fig. 1).

A significant reduction in the mean N-D difference after surgery was achieved in both groups: 5.4 PD preoperatively to 2.5 PD postoperatively at the last follow-up in ULR (*p* = 0.001) and 8.2 PD to 2.4 PD in RR (*p* = 0.001). However, the postoperative mean reduction amount in the N-D difference was not statistically significantly different between the two groups from 1 month to the last follow-up (*p* > 0.05), except at 3 months (*p* = 0.022), where the ULR group achieved significantly lower results (4.4 ± 2.8 PD) than the RR group (6.8 ± 3.4) (Table 3).

**Table 3:** Mean reduction in near-distance difference (PD) after the surgery.

	ULR group (n = 30)	RR group (n = 27)	*p* value*
**Postoperative**			
1 month	4.8 ± 3.1	6.8 ± 4.6	0.181
3 months	4.4 ± 2.8	6.8 ± 3.4	0.022
6 months	3.9 ± 3.5	6.0 ± 4.5	0.241
12 months	2.4 ± 4.0	5.0 ± 5.3	0.115
Last follow-up	2.9 ± 4.4	4.4 ± 4.9	0.298

Mean ± standard deviation.

*PD* prism dioptres, *ULR group* patients who underwent unilateral lateral rectus recession. *RR group* patients who underwent unilateral lateral rectus recession-medical rectus resection.

*Mann–Whitney U test.

There was no significant difference in the success rate between groups at every postoperative visit: 63.3% in ULR and 70.4% in RR at the last follow-up (*p* = 0.574, Table 4). Four patients (14.3%) in the ULR group and 21 (77.8%) in the RR group complained of diplopia due to esodeviation at postoperative 1 day. Among them, no patient in the ULR group complained of diplopia from 1 week postoperatively. In contrast, in the RR group, diplopia persisted in 12 (44.4%) at postoperative 1 week, two (7.7%) at 1 month, one (5.3%) at 3 months and none after 6 months. There was no patient with manifest esotropia or esophoria at the last visit (mean follow-up duration: 30.2 months in ULR, 30.0 months in RR) in the both groups.

**Table 4:** Surgical success rates (%) in ULR and RR group.

	ULR group (n = 30)	RR group (n = 27)	*p* value*
**Postoperative**			
1 month	96.4	92.3	0.604
3 months	87.5	95.2	0.611
6 months	68.2	85.7	0.281
12 months	80.0	73.3	0.666
24 months	40.0	83.3	0.074
Last follow-up	63.3	70.4	0.574

Surgical success = an alignment between 10 PD of exodeviation and 5 PD of esodeviation at distance and near.

*ULR group* patients who underwent unilateral lateral rectus recession, *RR group* patients who underwent unilateral lateral rectus recession-medical rectus resection.

*Fisher’s exact test.

Lateral gaze incomitance was presented in five patients of the ULR group (16.7%) at postoperative 1 day, which disappeared from 1 week visit in one patient, lasted until postoperative 1 week in three patients and 6 months in one patient, whereas none of the patient in the RR group had lateral gaze incomitance postoperatively. Three patients in the ULR group were performed reoperation for recurrent exotropia at 25, 45, and 118 months, respectively, from the first surgery, and 2 in the RR group at 54 and 62 months.

## Discussion

As we are not aware of any reports on the outcome of ULR for CI-type IXT, we compared ULR and RR in patients with CI-type IXT. We found that ULR was as effective as the RR procedure in treating CI-type IXT in terms of collapse of the N-D difference and the surgical success rate.

The reference value of N-D differences used in the classification remains somewhat controversial across studies: Burian and Spivey^1,8^ recommended a 10-PD difference, which has generally been used till date. However, Hardesty et al.^2^ used a 5-PD difference as the reference value, and another study used a 15-PD difference^3^. While Suh et al.^20^ used a 10-PD as the reference value in distance exodeviations of more than 30 PD, in exodeviations of less than 30 PD, the value was defined as one-third of the distance deviation because 10 PD would be a relatively significant difference in a small angle, and the effects of treatments on the change in types might be underestimated in such cases^20,21^. In this study, based on Hardesty et al.’s classification, the CI type was defined as exodeviation that was 5 PD larger at near than at distance fixation because only patients with IXT of small to moderate angles, even at near fixation, were included to analyze the surgical results of one muscle surgery (unilateral lateral recession).

CI-type IXT is much less common than other types of IXT and has been reported to occur in only 2.8‒4.2% of the IXT^22,23^. Many surgical methods have been used to treat CI-type IXT, but the surgical outcomes are variable and mostly unsatisfactory, with success rates ranging from 18 to 92%^1,6,8–11^.

Under the assumption that MR has the main effect on the near deviation angle and LR plays a role mainly in distance, MR resection has been classically introduced for the treatment of CI-type IXT. However, the success rates of unilateral or bilateral MR resection(s) for CI-type exotropia have been reported to range from 27 to 67%, which has motivated the development of new surgeries^6,17,24–26^. Choi and Rosenbaum^6^ performed unilateral or bilateral MR resection with an adjustable suture in 21 consecutive patients with CI-type IXT. The surgical success rate (10 PD esodeviation to 10 PD exodeviation) was 76.2% at the last examination. In 1995, Kraft et al.^5^ introduced the revised method for RR for the CI-type X(T) in which LR recession and MR resection were biased to the distance and near deviations, respectively, and MR was strengthened more than LR was recessed. They reported that this surgery had a low risk of creating long-term postoperative esodeviations at distance. In Wang et al.’s^9^ prospective study, the surgical results of the revised RR were better than those of unilateral or bilateral MR resection(s), reducing distance and near deviation. However, a high proportion of the patients experienced early postoperative overcorrection.

Raab and Parks reported that correction of N-D exotropia was obtained in only 28% of patients at 6 months after bilateral LR recession^27^. Bilateral LR recession augmented to near exodeviation was described in the study by Farid and Abdelbaset^17^. In this study, the success rate in the ULR group was 63.3% at the last follow-up, which was higher than that achieved in previous studies of bilateral LR recession: 40% in Raab and Parks’s study^27^ and 50% at distance and 27.2% at near in Farid and Abdelbaset’s study^17^ after bilateral LR recession.

In our study, the postoperative exodeviation and N-D difference were significantly reduced in both groups; however, there was no statistically significant difference between the ULR and RR groups. The exact mechanism of N-D difference reduction has been unclear. It seems that after certain amounts of muscle surgeries for strabismus, the same amount of the angles of near and distance exodeviation may not be corrected, but the preoperative greater angles (near exodeviation in cases of CI-type exotropia) may be more corrected than the smaller angles (distance deviation in CI-type exotropia). Therefore, a significant reduction in the mean N-D difference was achieved after ULR as well as RR procedures.

In addition, success rates were not significantly different between the two groups, and risk of postoperative esodeviation or diplopia at distance after ULR was lower than that after RR. Moreover, in this study there was no complaint for the lateral gaze incomitance or diplopia from 6 months after ULR., which may be a concern when performing only LR recession without MR resection. However, considering these concerns, clinicians need to performed a large amount of ULR with caution.

This study had several limitations. First, because of the retrospective study design, the surgical method was selected without any specific policy, although the surgeon had no preference for either the ULR or RR procedure. Second, the preoperative near exodeviation angles and N-D difference in the ULR group were smaller than those in the RR group; thus, minor bias could have occurred. However, the collapse of the N-D difference after ULR was achieved with results similar to those obtained after RR, and there was no statistically significant difference between the two groups. Further studies should be conducted with a prospective, randomized design with a larger number of patients to confirm the efficacy of ULR in patients with CI-type IXT.

Notwithstanding these limitations, this study is meaningful in that it provides data suggesting a favorable outcome of unilateral LR recession in CI-type IXT, with comparable results to those of the recess-resect procedure. In CI-type IXT measuring less than 25 PD, there was no significant difference in the amount of N-D difference reduction after surgery or the surgical success rate between ULR and RR.

In conclusion, unilateral LR recession for IXT with CI measuring less than 25 PD is a useful surgical procedure because it produces surgical outcomes similar to those of the unilateral RR.
